# Codon based co-occurrence network motifs in human mitochondria

**DOI:** 10.1038/s41598-018-21454-2

**Published:** 2018-02-15

**Authors:** Pramod Shinde, Camellia Sarkar, Sarika Jalan

**Affiliations:** 1 Centre for Biosciences and Biomedical Engineering, Indian Institute of Technology Indore, Simrol, Indore, 453552 India; 2 Complex Systems Lab, Discipline of Physics, Indian Institute of Technology Indore, Simrol, Indore, 453552 India

## Abstract

The nucleotide polymorphism in the human mitochondrial genome (mtDNA) tolled by codon position bias plays an indispensable role in human population dispersion and expansion. Herein, genome-wide nucleotide co-occurrence networks were constructed using data comprised of five different geographical regions and around 3000 samples for each region. We developed a powerful network model to describe complex mitochondrial evolutionary patterns among codon and non-codon positions. We found evidence that the evolution of human mitochondria DNA is dominated by adaptive forces, particularly mutation and selection, which was supported by many previous studies. The diversity observed in the mtDNA was compared with mutations, co-occurring mutations, network motifs considering codon positions as causing agent. This comparison showed that long-range nucleotide co-occurrences have a large effect on genomic diversity. Most notably, codon motifs apparently underpinned the preferences among codon positions for co-evolution which is probably highly biased during the origin of the genetic code. Our analysis also showed that variable nucleotide positions of different human sub-populations implemented the independent mtDNA evolution to its geographical dispensation. Ergo, this study has provided both a network framework and a codon glance to investigate co-occurring genomic variations that are critical in underlying complex mitochondrial evolution.

## Introduction

“Nature’s stern discipline enjoins mutual help at least as often as warfare. The fittest may also be the gentlest.” as proclaimed by Theodosius Dobzhansky on mankind evolution, like galaxy formations, the evolution of human remains the generation long grit of thinkers. Non-recombining loci, such as the maternally inherited mitochondrial DNA have been known to provide a richer estimation insight into ancestral human genetic variations during evolution^1^. Mitochondrial DNA is highly polymorphic, and its diverse nature in humans may contribute to individual differences in function^2,3^. Sequence variations considered in human mtDNA are selectively neutral^4^. Under this assumption, the pool of mutations that enter a population and are subsequently fixed by the selection or by the stochastic process of genetic drift will differ across populations^5,6^. Conversely, mtDNA polymorphism might also have been shaped via positive selection because of mito-nuclear co-evolution^7^. The evidence in support of mtDNA sequence polymorphism affecting phenotypic variation in metabolism, life-history traits and fitness is compelling^8^.

In retrospect, several studies were carried out that highlighted the role of natural selection and genetic drift on the diversity and divergence of mtDNA^2^. The methods based on phylogenetics, distances among sequences and clustering have successfully classified various traits and migrational events in human population^9^. Furthermore, genome wide studies and mapping techniques, such as quantitative trait loci and linkage disequilibrium have effectively sorted genes and alleles among the population as well as a linked group of single nucleotide polymorphisms (SNPs), as a causing agent^8,10^. With the above advent of techniques, various probabilistic and machine learning based efforts have been made to measure the rate at which different types of mutation occur. Besides that these efforts have helped not only to predict quantitatively subsequent fate of mutations in populations but also to assess the way mutations affect some properties of population^8^. Mutation in mtDNA is attributed to its protein-coding part^11^ because a large portion of non-coding mtDNA is directly known to co-evolve with coding mtDNA^6,12^. Independent dispersal of evolutionary signatures in mtDNA and extent of their conservation in human sub-populations are critical to understand complex mitochondrial evolution and formation of genetic code^11,13^. Moreover, phenotypic variation particularly SNP must be interacting with one another, at least in the loose sense that both influence the same phenotype^14^. Essentially, tree based ensemble methods have been proposed for identification of SNP-SNP interactions^15^. In addition, SNP based enrichment and functional nodal mutation based approaches have effectively inferred ancestry^16^ and functional convergence in human population^17,18^, respectively. These methods have suggested that the detected statistical interaction is considered equivalent to the positive real interaction for downstream analysis^19,20^. In essence, the evolutionary behavior of the genome often involves co-operative changes in variable genome positions^18,21^ and results in various evolutionary genomic discernments. In this study, nucleotide co-occurrence were precisely captured to form network model.

**Figure 1:**
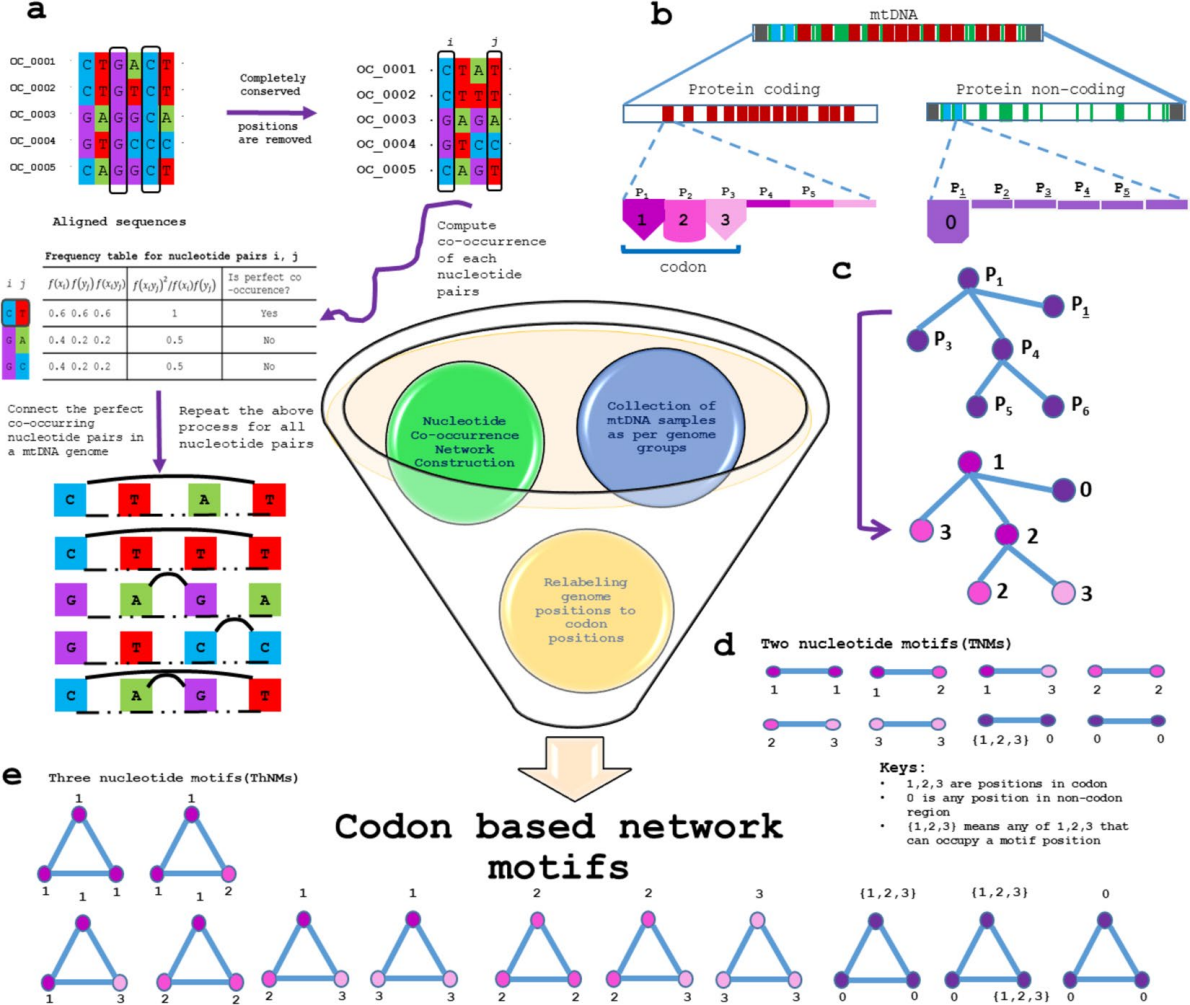
An overview of codon-based nucleotide co-occurrence network analysis. (**a**) Flow diagram depicts the schematic process of nucleotide co-occurrence network construction^28^. Nucleotide co-occurrence network for each genome was constructed (Supplementary Fig. S1), likewise there were 18,411 networks considered for the study. (**b**) Codon positions were identified concerning to corresponding nucleotide positions. (**c**) Genome positions were relabelled as the codon positions for each nucleotide co-occurrence network. For instance, *P*_1_ can be present in protein coding region which was relabelled as 1 whereas ${P}_{\underline{1}}$ can be present in protein non-coding region which was relabelled as 0. Likewise, the entire network was relabelled for the further analysis. (**d**,**e**) Display different types of two and three nucleotide motifs. Circle represents node and number associated with the circle represents codon position.

Complex network science revolves around the hypothesis that behavior of complex systems can be elucidated in terms of structural and functional relationships between their constituents by means of a graph representation^22^. Herein, nucleotide positions are network nodes and if they co-occur together they form an edge between the two nodes making entire nucleotide co-occurrence network (Fig. 1). Network framework provides a cue into whether the structural environment confers opportunities for or constraints on individual node action^23^. Furthermore, network motifs considered to be topologically distinct interaction patterns, are known to be a fundamental feature of networks and represent the simplest building blocks^24,25^. In biological networks, network motifs represent meaning to protein family and to pathway conservation^26^, which has been shown theoretically and experimentally^27^. Herein, we studied network motifs by relabelling nucleotide positions with codon positions. Despite the tremendous advancements in the field of network theory, very few approaches have taken nucleotide based genomic co-occurrences into consideration^28,29^, whereas codon based genomic co-occurrences have not been described previously. Genomic co-changes are well reported as a consequence of evolutionary events such as climatic changes, migration events, genetic traits etc^30^. In a way, co-occurrences of codon and non-codon positions among the genomes across sub-populations are expected to help in both gaining insights into co-changes in genomes and illustrating patterns of co-changes across sub-populations.

The primary aim of this study was to describe the level of diversity observed with respect to co-occurring mutations in the human mitochondrial genomes. The present study considered 18,411 human mitochondrial genomes, covering most of the mtDNA diversity of the global human population. Our analysis showed that variable nucleotide positions among mitochondrial genomes residing in different human sub-populations implemented the independent mtDNA evolution with respect to its geographical dispensation. The second aim was to provide a simple framework to investigate cooperative genomic interactions which are critical in underlying complex mitochondrial evolution. Construction of nucleotide co-occurrence networks with respect to different human sub-populations provided fundamental insights into genetic code in terms of codon bias evolution that we studied in depth using analysis of network motifs. Finally, the third aim was to compare the observed levels of diversity in each genome group making use of codon bias and network tools. Network motifs based on codon positions gave the evolutionary signature of association between codon positions present within and among human sub-populations. Revealing importance of each codon position in mitochondrial evolution, our codon-based network motif analysis have yielded evolutionary preferences of codon positions in the formations of lower and higher order motifs.

## Results

### Statistics of Variable sites

#### Variome is well conserved and independently maintained

**Figure 2:**
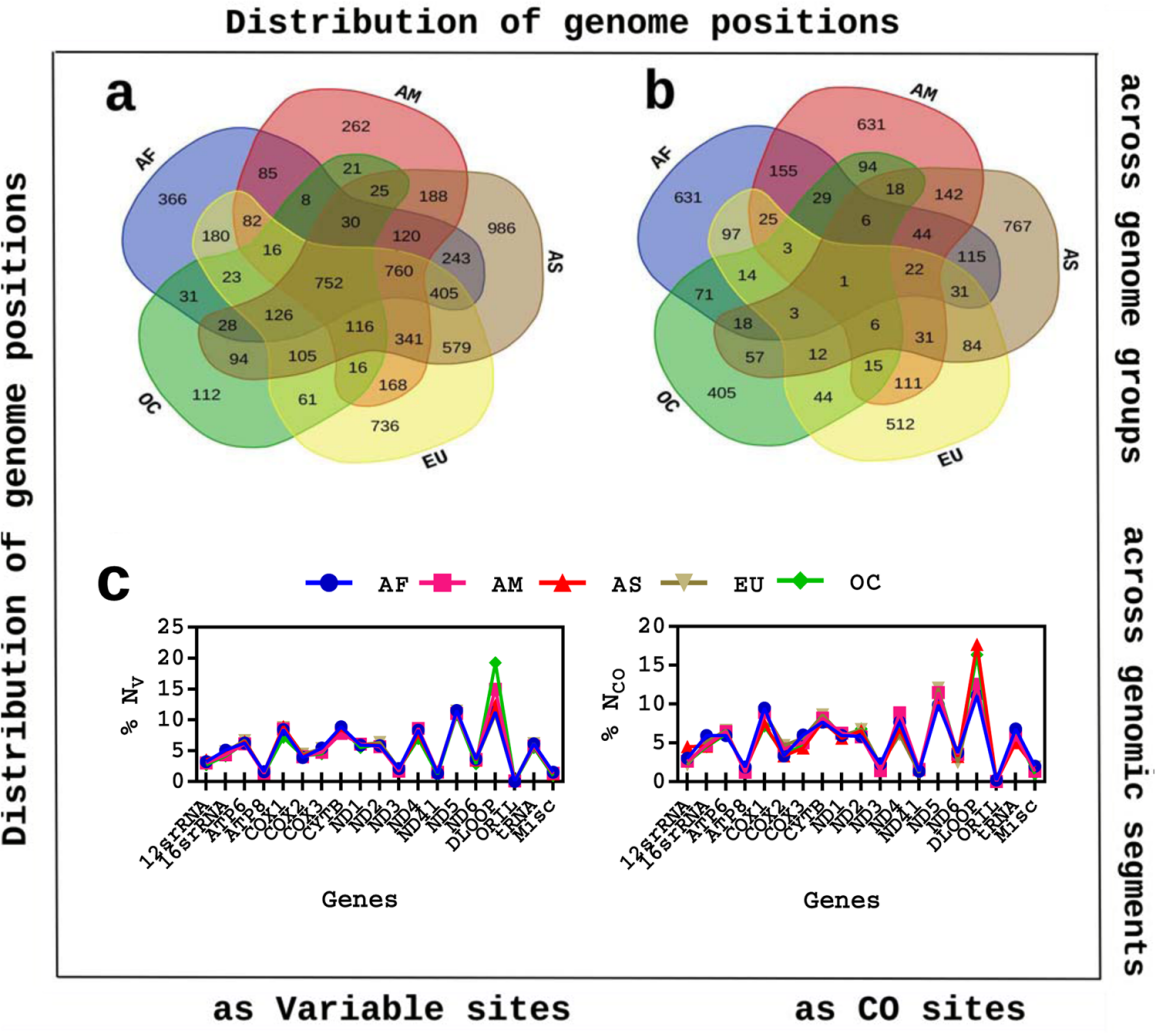
Distribution of variable and CO sites across genome groups and genomic segments. (**a**,**b**) Variable sites and CO sites occurred at similar genome positions in different set of genome groups such as two, three, four and all five. Additionally, certain variable sites were only occurred at a particular genome group. (**c**) Percentage of V (left panel) and CO (right panel) occurred in both protein-coding and non-coding genomic segments of mitochondrial genome. There are total 22 tRNA genes present in human mitochondria, all tRNA segments were termed as one tRNA to find their collective role. Counts of both V and CO showed similar patterns of increase and decrease.

As a first step towards tracing co-occurring variable site, mtDNA sequences were aligned and analyzed among each genome group. A total of 17,174 variable sites were screened from all the genome groups that would make a total of 7065 unique variable sites out of 16,881 bp genome size. In other words, 42% nucleotide positions of human mtDNA are incidentally at least once mutated till date. Variome sizes (*V*) for each genome groups were different (Table 1). In addition, a large portion of variome in each genome group was found to occur at similar genome positions (Table 1 and Fig. 2b). Particularly, a significant portion of variomes (21%) were found to be common among five genomes groups (Fig. 2b), indicating the major part of geographical variomes are same across the human population. Secondly, there was a pattern observed in common variable sites among different genome groups. The largest portion of variomes commonly occurred among {*AS*, *AM*, *AF*, *EU*}, {*AF*, *AS*, *EU*} and {*AM*, *AS*, *AF*} genome groups (Fig. 2b), indicating that human population habitating in AF, AM, AS and EU geographical regions have more number of common variable sites as compared to OC geographical region. It also turned out that AS and EU genome groups share the highest number of variable sites. There are weak evidence that the level of evolution in mitochondria is correlated to the effective population size^8^ and above results suggested that genotypic changes in human mtDNA are more resident into the Asian and European geography^31^, which is well known.

**Table 1:** Data and network statistics.

Genome group	*S*	*N*_*V*_	〈*N*_*Co*_(±)〉	〈*E*(±)〉	〈*k*〉
Africa (AF)	2323	3255	914 (5)	967 (6)	2
America (AM)	1692	2990	967 (4)	1211 (5)	2
Asia (AS)	5715	4898	426 (5)	474 (19)	2
Europe (EU)	7142	4466	470 (5)	334 (8)	2
Oceania (OC)	1539	1564	455 (2)	473 (1)	2

*S* represents sample size or number of genomes, *N*_*V*_ represents number of variable sites or size of variome. 〈*N*_*Co*_〉 and 〈*E*〉 represent average number of nodes and average number of edges across networks of a genome group, respectively whereas 〈*k*〉 represents average degree of the networks of a genome group.

Moreover, mtDNA possessed more sequence diversions among some genome groups as compared to others as shown in Fig. 2a. Counts of common variable sites corresponded to common evolutionary mechanisms among a set of genome groups whereas counts of group specific variable sites corresponded to evolutionary mechanisms pertaining to a particular genome group. Variable sites introspect more robust functionalities to the mitochondrial genome and provide both genotypic polymorphism and associated phenotypic variation^32^ which was perceived by a different set of variable sites among each genome groups. Overall, molecular sequence based diversions in terms of SNP were very well conserved across genome groups as well as independently maintained within genome groups.

#### Co-occurring variable sites show region specific variations

In the next step towards identifying co-occurring variable sites which were thought to be co-evolved^28^, the pairs of genome positions were screened with co-occurrence frequency (*C*_*f*_) equal to 1. It should be noted that a small portion of variable sites participated as perfect co-occurring variable (CO) sites that constituted nodes of the network (Table 1). Unlike variable sites, CO sites did not share more number of sites in common among genome groups (Fig. 2a,b). Furthermore, distribution of V and CO sites showed similar patterns in increase and decrease among individual genomic segments and among all genome groups (Fig. 2c), impelling nucleotide positions were conserved whether nucleotide positions participate as co-occurring variable sites or not. V and CO sites were possessed by both protein coding and non-coding regions (Fig. 2c). These results not only urged that SNP can occur throughout mtDNA but also highlighted that these population genomics signatures can be widely conserved among all genomic segments, including both the coding and non-coding segments, across genome groups. Regional variations accumulate mtDNA diversity purely attributed to genetic drift in each genome group^2,33,34^ and such region specific variations also found to persist in resulting co-occurrence of sequence based variations. Similarity in patterns among genomic segments and genome groups should point towards the majority of literature that has supported neutral evolution in human mtDNA^8^.

#### CO pairs display intra- and inter- DNA adaptation

**Figure 3:**
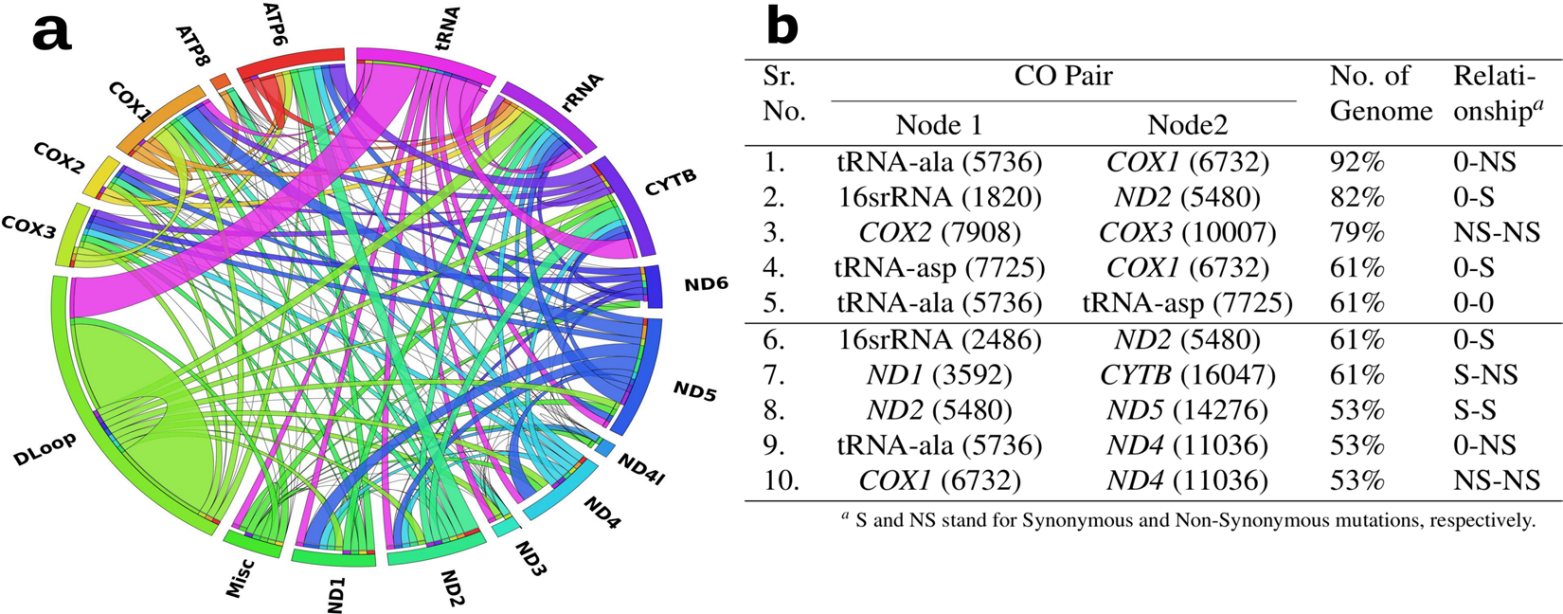
Statistics of CO pairs. (**a**) Circos plot illustrates the shared CO pairs among protein-coding gene, non-coding, rRNA and tRNA regions. Links or ribbons represent the frequency of CO pairs between two genomic segments. D-Loop has maximum number of intra- genomic segment CO pairs. (**b**) Table of predominant CO pairs existing among all genomes showing CO pairs occurred between genome positions *i*.*e*., node 1 and 2 and hence have represented association between two genome positions (shown in associated braces). Codon positions represented the relation among different types of mutations *i*.*e*., synonymous (S) and non-synonymous (NS) mutations. Mutations among non-codon regions can also be termed as S mutations^51^, but to make it distinguished from codon position 3 based mutation, genome sites at non-codon regions were considered as 0. The predominant CO pair associations were (i) S-NS, S-S, NS-NS types *i*.*e*., among protein-coding genes, and (ii) S-0 and NS-0 types among protein-coding gene and non-coding regions.

Analysis of CO pairs provided an essential understanding of the relationship between two independent genome locations (Fig. 3). Genetic adaptation in response to selection on polygenic phenotypes may occur via subtle allele frequency shifts at many loci or they are resultant of effector mutations at one or many places^35^. CO pairs can be formulated within a particular mitochondrial gene (intra-gene) or between two mitochondrial genes (inter-gene). We enumerated CO pairs and calculated the number of CO pairs formed across the length of mtDNA. Less number of CO pairs were formulated among intra-gene segments than inter-gene segment (Fig. 3a). It impelled the importance of much anticipated polygenic co-evolution among mtDNA genes. The relationship between correlated mutations and spatial proximity has not only been found among residues in the same protein but also among residues in different proteins^36,37^. Along these lines, CO pairs formulated among each intra- and inter- genomic segments (Fig. 3a) were counted. D-Loop has maximum CO pairs among its own intra-genomic segment whereas it has formulated the least number of CO pairs with rest of the genome (except tRNA). *COX1* have formulated the highest number of CO pairs with inter- genomic segments. Also, tRNA regions found to have much lesser (0.9%) intra-gene CO pairs, but at the same time they have formulated CO pairs with the almost all protein-coding genes. This observation should attend the corollary that evolution of tRNA genes is largely controlled by recipient *i*.*e*., protein-coding genes. Also, mutation restrictions between protein coding genes and tRNA reported to have consequence to form secondary structure conformation^38^.

Apart from tRNA regions, protein-coding genes viz. *ND5*, *COX1* also formulated CO pairs with almost all other genes (Fig. 3a). At the same time, *ND1* and *COX1* formulated the least number of intra-gene CO pairs. Overall, associations among nucleotide positions of single or multiple genes and individual genomic segments showed particular preferences to formulate CO pairs within and outside its genomic segment. These genomic associations can be corollary of different environmental effects, such as climatic and geography based events resulted into natural selection occurring in a particular genomic segment^39^. Both gene wide association and molecular structure based interaction studies have broadly shaded light on the fact^32^ and perfect nucleotide co-occurrence were presumed to provide insights into intra- and inter- genomic segment adaptation.

Moreover, it would be interesting to look for functional insights into how an individual CO pair was formulated. In order to investigate this, predominant CO pairs were enlisted which were found in atleast two genome groups. Such top 10 CO pairs were picked up to further understand their biological insights (Fig. 3b). These CO pairs were formed among inter-genomic segments and were found between protein-coding genes (e.g., *COX2*: *COX3*, *ND1*: *CYTB*, *ND2*: *ND5*, *COX1*: *ND4*) and between protein-coding gene and non-coding gene regions (e.g., *COX1*: tRNA, *ND4*: tRNA etc). These short-listed pairs would be evolutionarily more conserved since these predominant pairs have co-occurred in maximum number of genomes across genome groups and were thought to provide genotypic insights in terms of what mutation did they encode for. On the basis of relation between codon positions and type of mutations displayed by predominant CO pairs (Fig. 3b), it was possible to say that there can be any mutational preferences to formulate CO pair between protein-coding and non-coding gene regions and between different codon pairs (see legends of Fig. 3b). However, it was interesting to observe that most of the CO pairs found in atleast two genome groups, were formed either among protein-coding genes or between tRNA and protein-coding gene, implementing the selective role of coding DNA to form CO pairs (illustrated in SM).

### Co-occurrence networks exhibited same average connectivity

Nucleotide co-occurrence network was constructed for each genome, where all variable sites forming CO pairs constituted the nodes, and the edges represented co-occurring nucleotide positions. Network size (*N*) was found to be the same within each genome group (Table 1) which was expected since these genomes have high sequence similarities and they differed only at variable genome sites. Though genomes among genome groups also have high sequence similarity, still *N* was found to be different which was intuitive since *N* is depended upon a number of CO pairs formed in each genome group (Table 1). Furthermore, average connectivity (〈*k*〉) was calculated for each network that would represent the average number of co-varying partners per nucleotide position. Surprisingly, 〈*k*〉 for each co-occurrence network was found to be the same irrespective of genome groups (Table 1). Though co-evolution is an economic evolutionary mechanism^36,40^, still co-evolution of nucleotide positions were widely conserved across all mitochondrial co-occurrence networks. In following, we studied on how different codon positions were associated with identification of network motifs for 13 mtDNA protein-coding genes *i*.*e*., codon regions and in like manner we extended our understanding by discerning network motifs among codon regions and other parts of the genome *i*.*e*., non-codon regions.

### Codon positions make network motifs

**Figure 4:**
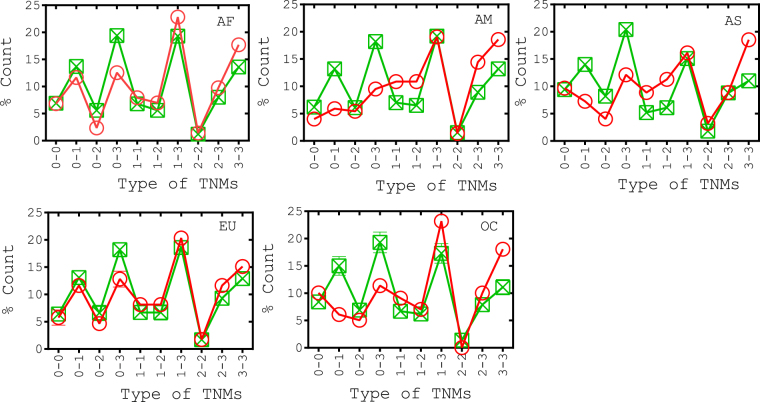
Distribution of TNMs in different genome groups. These motifs were made up of different codon positions, such as 1, 2 and 3, whereas 0 in motif have represented the nucleotide position of non-codon region. There were total 10 possible TNM connected patterns in which 0-0 can be found among non-codon regions; 0-1, 0-2 and 0-3 can be found between codon and non-codon regions whereas remaining TNMs can be found among codon regions. Circles and squares denote motif counts in the nucleotide co-occurrence and the randomized networks, respectively. 10,000 realizations were performed to construct the randomized networks for each genome group.

**Figure 5:**
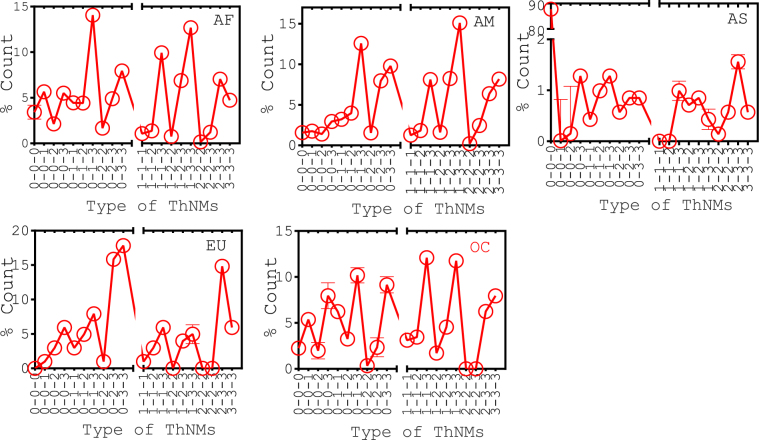
Distribution of ThNMs in different genome groups. There were total 20 possible ThNM patterns enlisted. Circles denote motif count in nucleotide co-occurrence networks. Randomized networks have shown significantly lesser number of ThNMs (Supplementary Fig. S2).

Network motifs of order two and three were calculated. Codon positions in each motif share common biological functions suggesting that a particular codon position would have the same sensitivity across the length of the genome. For instance, codon position 1 would possess high sensitivity irrespective of codon placement at any location in the genome. Network motifs considered here were the combination of synonymous (S) and non-synonymous (NS) mutations. Particularly, we enlisted 10 two order and 20 three order possible motif types. Motifs count found as large as in the real nucleotide co-occurrence network occurred very rarely in that of corresponding randomized networks (Figs 4 and 5). The significant finding was that region-specific interactions among motifs did exist and that they seemed to split-up the group of codon positions into biologically meaningful clusters.

#### Two nucleotide motifs (TNMs)

TNMs among codon region have appeared at different frequencies than expected at random, suggesting that network motifs would be of specific biological functions and formation of TNMs were not merely random. This observation also provided an ascendancy of codon positions in the motif formation. Curiously, most of the TNMs were formulated where codon position 3 was acting as one of the motif nodes (Fig. 4), overtoning the influence of codon position 3 in the motif formation. Codon position 3 motifs have different counts than those of the corresponding randomized networks (Fig. 4), which clearly implicated an evolutionary preference given to motifs with codon position 3 in the codon regions. These less sensitive codon sites favored co-mutations, perhaps deliberately, with all codon sites as given by a larger count of S-S associations (0-3 and 3-3) and NS-S (1-3 and 2-3) associations.

Moreover, high sensitive codon sites less favored to co-mutate among themselves as shown by a lesser count of NS-NS (1-1, 1-2 and 2-2) associations. Apart from that codon position 2 favored to co-mutate selectively with other codon positions including itself (2-2), whereas codon position 1 favored to co-mutate omnivorously with all codon and non-codon positions. In addition, NS-NS co-mutations were found in lesser number than S-S co-mutations, implementing the most-sensitive codon sites have exhibited a lesser preference to co-mutate among themselves as compared to those of the least-sensitive codon sites (0-0, 0-3 and 0-3) in short-range conservations. Interestingly, non-codon region has lesser counts of homogeneous motifs (0-0) than heterogeneous motifs (0-1, 0-2 and 0-3) (Fig. 4). In other words, non-codon region favoured more number of short range co-mutations with codon regions. In line particularly, TNMs were screened among non-codon regions *i*.*e*., tRNAs, rRNAs and D-Loop regions. Large part of heterogeneous motifs found between RNA region and coding region, whereas most of homogeneous motifs found among intra- D-Loop regions (Table 2). D-Loop and RNA regions are known to be the signatures of population genomics variations in most of the mtDNA associated studies^41^ and similar fact was observable in the co-associations of each non-codon genomic segments.

**Table 2:** Pairwise associations found among all networks and TNMs.

Genome group		CDS^*a*^:CDS	CDS:D-Loop	CDS:Misc	CDS:*ORIL*	CDS:rRNA^*b*^	CDS:tRNA	D-Loop:D-Loop	D-Loop:Misc	D-Loop:*ORIL*	D-Loop:rRNA	D-Loop:tRNA	Misc:Misc	Misc:*ORIL*	Misc:rRNA	Misc:tRNA	*ORIL*:*ORIL*	*ORIL*:rRNA	*ORIL*:tRNA	rRNA:rRNA	rRNA:tRNA	tRNA:tRNA	Total^*c*^
Africa (AF)	Net-work	594 (1)	0 (1)	27	3	158 (1)	115 (1)	18 (5)	0 (1)	0	0 (0.2)	0 (0.1)	7 (2)	0	3	4	0	0	0	11	18	9	967 (6)
America (AM)		835 (2)	0 (1)	19	0	174 (1)	138	9 (2)	8 (2)	0	0 (0.1)	0 (0.2)	6 (1)	0	1	2	0	0	0	6	11	4	1212 (5)
Asia (AS)		187 (5)	0 (1)	8	0	37 (1)	38	184 (21)	0 (2)	0	0 (0.1)	0 (0.1)	6 (2)	0	2	1	0	0	0	9	0	2 (1)	474 (24)
Europe (EU)		217 (6)	0 (0.2)	8	0	47 (4)	49	3 (1)	0 (0.1)	0	0 (2)	0 (5)	0 (0.4)	0	0 (0.3)	1	0	0	0	5	3	1	334 (8)
Oceania (OC)		308 (3)	4 (2)	9 (1)	0	57 (2)	63 (1)	6 (1)	0 (0.2)	0	1 (1)	0 (0.2)	0	0	1 (1)	2	0	0	0	5 (1)	11 (1)	6	473 (8)
Africa (AF)	TNM	143 (1)	0	2	1	33	21	6 (2)	0 (1)	0	0	0	1 (1)	0	0	0	0	0	0	3	2	3	215 (2)
America (AM)		166 (1)	1	3	0	19	24	5 (1)	0	0	0	0	0 (0.3)	0	1	0 (0.2)	0	0	0	3	0	0	221 (2)
Asia (AS)		83 (2)	0 (0.2)	2	0	15	12 (2)	8 (1)	0	0	0	0 (0.1)	0	0	0 (0.1)	0	0	0	0	3	0	1 (1)	124 (5)
Europe (EU)		112 (3)	0 (0.1)	2	0	23 (4)	25	3 (1)	0 (0.1)	0	0 (2)	0 (0.1)	0 (0.4)	0	0 (0.3)	0 (0.1)	0	0	0	3	3	1	172 (5)
Oceania (OC)		67 (1)	4	2	0	5	11 (1)	6 (1)	0	0	1	0 (0.1)	0	0	0	0	0	0	0	1	1	1	99 (2)

Pairwise associations are enlisted for each genome group by taking the average of all networks and value in braces is the standard deviation. A larger portion of pairwise associations were formed among protein coding region (CDS), and vice versa were observed in the non-coding region (except in AS). Apart from that significant number of co-occurring pairs were observed between CDS and RNA (both tRNA and rRNA). Count of TNMs was less as compared to ThNMs when total pairwise associations among network and corresponding TNMs were compared (Supplementary Fig. S3). ^a^Coding DNA Sequence composed of all 13 protein coding genes. ^b^Both 12s rRNA and 16s rRNA. ^c^Total actual counts of co-occurring pairs and TNMs. Average was taken over all networks in a genome group.

#### Three nucleotide motifs (ThNMs)

We detected locally dense regions in the network that were thought to be evolutionarily conserved. There have been several different approaches to identify clustering based network motifs and these studies have provided distinct outcomes with respect to the resulting network motifs^42^. The main conclusion, however, was that nucleotide co-occurrence networks tend to be locally connected^28,29^, whereas network motifs were entirely separable from the rest of the network. In fact, identified complete sub-graphs were nested within each other.

ThNMs in genome networks were found to be significantly higher than that of the randomized networks, which was not surprising. However, the interesting result was that most of the ThNMs were formed when atleast one node of the motif has belonging to the codon region (Fig. 5), clearly asserting the importance of codon positions in long-range conservations. In addition, it would be intriguing to study how codon positions co-mutated amongst and with other codon positions to devise long-range conservations. Foremost, the predominant ThNMs were the motifs formulated with the combinations of codon positions 1 and 3. In particular, 1-3 was the predominant two order motif in formulating ThNMs with all the codon and non-codon positions. This observation was consistent with TNMs analysis where 1-3 has the highest count. Furthermore, the role of homogeneous TNMs were analysed in formulating ThNMs. Motif 1-1 favoured to formulate ThNMs with all the codon and non-codon positions where the highest number of ThNMs were formulated with codon position 3 and the count of 1-1-1 was negligible. Motif 2-2 showed very selective role in formulating ThNMs with codon and non-codon positions whereas hardly any ThNMs were formulated with codon position 2 at all three positions of triplet. Motif 3-3 formulated ThNMs omnivorously with all codon and non-codon positions.

Interestingly, ThNMs formulated in very less amount when all three positions of triplet were occupied by the sensitive codon positions, such as codon positions 1 and 2. However, increase in ThNMs counts were observed when codon position 1 and 2 occupied at any two positions of triplet. In other words, the sensitive codon positions less favoured to co-mutate with themselves while forming higher order motifs. In addition, heterogeneous triplets between codon and non-codon regions were enumerated and found that 0-1-2, 0-2-3 types were the prominent heterogeneous triplets. Further, we analysed on how these heterogeneous TNMs were associated with codon positions. Both 1-2 and 1-3 motifs have more preference to form a higher order motifs with codon positions 2 and 3 than that of the codon position 1. Similarly, motif 2-3 have shown preference to form higher order motifs with codon positions 1 and 3 than that of the codon position 2. Overall, particular preferences were assigned with each heterogeneous codon position pair to form higher order motifs.

Moreover, ThNMs involving non-codon positions were analysed and it was observed that the count of 0-0-0 ThNMs was less, the result was consistent with the lesser count of 0-0 motifs (except in AS). In other words, a lesser preference was given to formulate motifs in non-codon regions. Also, more number of motifs in non-codon regions formulated with co-association of codon positions, incriminating the preference of codon positions to get co-evolved with non-codon region of mtDNA in long range conservations. Interestingly, most of ThNMs in AS genome group were 0-0-0 motifs (88%) and these 0-0-0 motifs were formulated among intra-D-Loop and among intra-miscellaneous genomic segments. In line, all the non-codon associations were screened among TNMs and complete networks. Non-codon associations favoured among individual intra- D-Loop and intra- RNA (e.g., tRNA: rRNA) regions than inter- D-Loop and RNA regions (e.g., D-Loop: tRNA) in all genome groups (Table 2). Overall, the known population genomics signatures such as D-Loop, RNA region^41^ less favoured to co-mutate with other part of non-coding segment but within themselves.

## Discussion

In this study, we have developed a network model based on pairs of co-occurring nucleotides over the length of the genome. The network representation of global human mitochondria genomes have provided a new perspective on mutation restrictions represented in terms of mutual mutations. All protein-coding genes, tRNAs, rRNAs and the non-coding portion of mtDNA took part in the formation of genomic co-occurrences. In particular, protein-coding and RNA genes were essential agents to formulate functional genomic co-occurrences. Furthermore, the characterization of the diversity present in five genome groups could serve as a comparative tool for intra-species evolution^43^. This comparison clearly showed the presence of heterogeneity in both variable sites and motifs across genome groups. More curiously, conceding coding DNA is the driver of genome evolution, codon-based motif analysis have demonstrated different co-occurrence preferences among codon positions in genome evolution.

The analysis of the co-occurring variable sites have resulted in uneven distribution of mutual mutations among each genome group, implementing the independent evolution of mitochondrial genomes. Besides that distribution of V, CO, and motif at both codon and non-codon region showed similar patterns when we compared them across genome groups. These observations clearly pointed that human mitochondrial genome must have undergone substantial levels of neutral selection, but some of the results were inconsistent and displayed biases towards forming co-evolutionary patterns. First of all, the least count of ThNMs within non-codon regions was observed whereas the number of ThNMs was higher with the involvement of codon positions. Codon positions demonstrated the selective role of mtDNA protein-coding genes in establishing long-range genomic associations which were poorly presented by non-coding region. Second, least-sensitive codon positions have a higher co-mutational ability as they were found to be over-represented throughout the analysis. Though codon position 3 has favored the largest count of co-mutations, it showed differential co-mutational preferences in each genome group. Such a differential codon bias ability of human mtDNA have earlier reported shaping human interspecific divergence^38^ which was also demonstrated by codon position 3 motifs. Third, codon position 1 and 3 were putative agents in forming co-mutations, in which the first one is highly sensitive and later is the least sensitive. Higher co-mutation ability between two codon positions indicated that alteration of amino acid and codon stability can be mutually favoring genomic activities^44^. Fourth, sensitive codon positions displayed the specificity to formulate motifs strictly in association with codon position 3 and non-codon positions. Conversely, this observation was also supported by lesser counts of motifs between codon position 1 and 2. Finally the fifth, mitochondrial evolution witnessed larger count of ThNMs over TNMs in which ThNMs formation was mainly attributed by codon positions where the formation required a group of three codon positions to co-evolve together which might be resultant of intense selection. Overall, these results have shaded light on the particular act of evolutionary mechanisms on each codon position to get differentially associated with another part of the genome.

This study have provided a complete listing of the co-associations among mitochondrial variations in the human population. Apart from that each codon position has a significant role in co-mutations which essentially displayed different adaptation in each genome group. Because of these facts, our predictions were more relevant in relating admixture of codon and non-codon associations and have provided much-needed codon glance to genomic positions, a novel genome sequence based network approach to understand genome evolution.

In summary, we found evidence that mtDNA should have undergone substantial amounts of adaptive evolution at the point of the network model. These results have important implications for human mtDNA evolution because both the diverse nature of co-mutational mechanisms and the diversification of human population certainly have upper hand to deliver efficient codon bias usage system in mtDNA^38,45^. Further, it would be interesting to extend the observations of the current study in comparison of human mtDNA with those of primates and other mammals where codon usage is a variant at both inter- and intra- specific level. Our approach can be readily generalized to any nucleotide co-occurrence network to improve on an evolutionary understanding of codons and their associations across prokaryotic as well as eukaryotic genomes.

## Methods

### Genome sequences

All genome sequences were retrieved in fasta file format from the Human Mitochondrial Database (Hmtdb)^46^, which provides a comprehensive, integral and non-redundant set of mtDNA genomes from geographically diverse human populations^47^. The ascertainment set comprised of 18,411 genome sequences from the five world continents, including 2323 African, 1692 American, 5715 Asian, 7142 European and 1539 Ocean genomes. Each of this continent was termed as a genome group.

### Construction of mitochondrial nucleotide co-occurrence networks

For each of these five sub-population genome datasets, the nucleotide co-occurrence network were constructed in which nodes represented as nucleotides at specific positions in the genome sequence and edges between nodes represented as co-occurring nucleotide pairs (Fig. 1) as following. (1) Since, the current study was based on the analysis of specific nucleotide position in the genome sequence, sequence data with end to end aligned were considered, maintaining each nucleotide position in genome sequence uniform. (2) All conserved nucleotide positions within samples of a genome group were removed, thus only variable nucleotide positions were left behind. A set of variable sites in a genome group was termed as variome. The count of variable sites (*N*_*V*_) is given in Table 1. (3) Using variable nucleotide positions, first the frequency of occurrence of all the nucleotide pairs *f*(*x*_*i*_*y*_*j*_) = *N*(*x*_*i*_*y*_*j*_)/*M* were calculated, where *N*(*x*_*i*_*y*_*j*_) denoted the number of co-occurrence pair (*x*_*i*_*y*_*j*_) at position (*i*, *j*) and *M* denoted total number of samples in a genome group. Second, the frequency of individual occurrence of single nucleotides *f*(*x*_*i*_) = *N*(*x*_*i*_)/*M* and *f*(*y*_*j*_) = *N*(*y*_*j*_)/*M* were calculated, where *N*(*x*_*i*_) and *N*(*y*_*j*_) denote the number of single nucleotides at their respective positions *i* and *j*^28^. (4) Co-occurrence of two nucleotides (*CO*) at position (*i*, *j*) was denoted as,

1 $$C{O}_{i,j}=\frac{f{({x}_{i}{y}_{j})}^{2}}{f({x}_{i})f({y}_{j})}$$

For perfect co-occurring variable sites, the adjacency matrix of the corresponding network was defined as,

2 $$\begin{array}{l}{A}_{{\rm{ij}}}=\{\begin{array}{ll}1 & {\rm{if}}\,C{O}_{i,j}=1\\ 0 & {\rm{otherwise}}\end{array}\}\end{array}$$

As each genome sequence had its own information of co-occurring nucleotide pairs, there were total *M* networks generated for each genome group.

### Network motifs based on codon position

All genome files were parsed to extract 13 polypeptide genes, 22 tRNAs, two rRNAs and three non-coding regions, such as D-Loop, *oriL* and miscellaneous (unclassified non-coding part), by aligning and comparing them with the standard reference sequences *i*.*e*., the revised Cambridge Reference Sequence (rCRS, GenBank id: NC_012920). rCRS has been used as the reference sequence to annotate mtDNA in molecular anthropology^48^. Furthermore, network motifs were defined based on the assignment of codon position to the genome position. As it has described in schematic Fig. 1b, the group of three genome positions in a gene region makes a codon. After construction of the nucleotide co-occurrence network, network nodes (genome positions) were relabelled with the position of codon. rCRS was referred in order to perform annotation of codon positions. In order to do so, codon position of each corresponding genome position was identified with respect to the reading frame of individual genes using their sequence position aligned with rCRS. In this way, each nucleotide position in protein coding gene regions (13 polypeptide genes) were relabelled with either 1, 2 or 3 and the rest of the genome positions *i*.*e*., non-codon positions were relabelled as 0. Two nucleotide (TNMs) and three nucleotide (ThNMs) network motifs were enumerated for each nucleotide co-occurrence network (see legends of Fig. 1). ThNMs or clique structures were identified using CFinder^49^ and TNMs were identified by subtracting nodes participating to form ThNMs from network adjacency list. After detailing all the motifs based on their positions in codons in different networks of each genome group, various statistical analysis of TNMs and ThNMs were performed.

### Generation of the randomized networks

For a stringent comparison, the randomized networks were generated with precise information of number of nodes and number of connections that of corresponding real nucleotide co-occurrence network. Construction of the randomized networks and their comparison with corresponding real networks allowed to estimate the probability that a randomized network with certain constraints has of belonging to a particular architecture, and thus assessed the relative importance of different network architecture. This information was collected by taking an average number of nodes (*N*) and average degree (〈*K*〉) of networks present in each geographical group. The randomized networks of size *N* and 〈*K*〉 were constructed using the Erdös Rényi random network model^50^ by connecting each pair of nodes with the probability (p),

3 $$p=\langle K\rangle /N$$

In this way, 10,000 randomized networks were constructed for each genome group. Further, TNMs and ThNMs for each real nucleotide co-occurrence network were enumerated and compared with those of the corresponding randomized networks.

## Electronic supplementary material

Supplementary Materials
