# Supplementary material for: Codon based co-occurrence network motifs in human mitochondria
Source: Sci Rep. 2018 Feb 15;8:3060. doi: 10.1038/s41598-018-21454-2 (PMC5814444; doi:10.1038/s41598-018-21454-2)
Supplement: Supplementary file 1 — Supplementary Materials [file 41598_2018_21454_MOESM1_ESM.pdf]

Supplementary Material

Codon based co-occurrence network motifs in human mitochondria

Pramod Shinde[1], Camellia Sarkar[1], Sarika Jalan[1,2*]

[1] Centre for Biosciences and Biomedical Engineering, Indian Institute of Technology Indore, Khandwa road, Simrol, Indore 453552, India.
[2] Complex Systems Lab, Discipline of Physics, Indian Institute of Technology Indore, Khandwa road, Simrol, Indore 453552, India.

Contents

Supplementary Information

Introduction
Evolution of variable sites
Evolution of co-occurring variable sites
Selective role of Coding DNA
Glossary
References

Supplementary Figures

Supplementary Figure S1: Schematic of nucleotide co-occurrence network.
Supplementary Figure S2: Frequency of each nucleotide among genome groups.
Supplementary Figure S3: Frequency of TNMs and ThNMs among genome groups.
Supplementary Figure S4: Distribution of ThNMs found in the randomized networks among different genome groups.
Supplementary Figure S5: Mitochondrial genome sample data processing.
Supplementary Figure S6: Schematic explaining the word collocations in the sentence.

Supplementary Tables

Supplementary Table S1: Genomic segments in human mtDNA.
Supplementary Table S2: List of genes with their associated protein names and UniProtKb Ids.

Supplementary Information

Codon based co-occurrence network motifs in human mitochondria

1 Introduction

Human mitochondrial genome contains 16,660 base pairs which encode for 37 genes, it lacks introns and there is one longer non-coding region, referred as control region [1]. Out of 37 genes, 13 polypeptide genes make enzymes involved in oxidative phosphorylation and remaining genes encode for tRNA and rRNA molecules (Supplementary Table S1). Numerous comparative genomics studies of human mitochondrial genomes have provided vital evolutionary evidences of anatomically modern human as well as diversification events which might have happened within human population across globe [2]. These robust evolutionary evidences (signatures) are understood as conservation of particular nucleotide positions in genomes, particularly single nucleotide polymorphisms (SNPs), copy number variations and indels [3]. Studies associated with these molecular signature have effectively identified SNPs, as well as independent evolutionary behavior of single gene. The evolutionary behavior of the genome often involves co-operative changes in these variable sites within and between genes. Analysis of correlated variable nucleotides in human mtDNA can be obscured by its evolutionary events, including conservation of these variable nucleotides across human sub-populations which are distributed in different geographical locations across the globe.

Co-occurrences of nucleotide positions between these genomes across sub-populations help in gaining insights into co-changes in genomes as well as conservation of these co-changes across populations. These co-changes are said to be largely based on climatic changes, migration events, personal as well as family traits etc [4]. Network studies based on co-changes have successfully given insights into many complex systems and have demonstrated that co-occurrences are essential ingredients in the evolution and are continuously adapting in contribution to evolutionary events. Studies associated with words related in the language organization have suggested that there are different words which are co-conserved at different parts of literatures and might reflect evolutionary as well as social history of lexicon [5,6]. There are different biological studies where different co-occurrences studied in depth. One of the biological network study associated with microbial co-occurrence relationships have demonstrated potential mechanisms of community organization throughout the human microbiome [7]. The study based on protein domain co-occurrence have provided insights into gene family evolution as well as functional association between different domain cores [8]. Further, domain co-association studies using genomics data have delivered in depth understanding into genome annotation for functional and evolutionary genome analysis [9, 10]. The epidemiological study with Ebola viral genomes could relate case fatality rate with properties of co-mutational networks [11]. Similar study with Influenza viral genomes have provided insights into human H3N2 virus evolution with co-operative changes into gene segments of virus [12]. Both of these studies with genomic co-occurrences of nucleotide positions in genomes of Influenza and

Ebola viruses also provide insights into how different gene regions are co-evolved in particular epidemic events of these viruses. In the current study, we consider 18,411 genomes of human mitochondria and identify variable sites and further construct networks of nucleotide position co-occurrences in genomes of different human sub-populations (Supplementary Fig. S4) as well as study the network motif structures in detail.

Network motifs are known to be fundamental feature of networks representing recurrent and statistically significant sub-graphs or patterns in networks [13, 14]. Motifs, considered to be topologically distinct interaction patterns in a network, may represent the simplest building blocks. In biological regulation networks, in which nodes are proteins and edges represent the interactions between them, each of the motifs has been shown theoretically and experimentally to perform a specific information-processing function [15]. Also in other biological networks such as protein-protein interaction networks [16] and metabolic networks [17], they represent meaning to protein family as well as pathway conservations. We identify network motifs based on codons in genome. These motifs give the signature of association between codons present within and between genes, are expected to provide an essential insights into codon position associations. A codon is defined as a sequence of three DNA or RNA nucleotides which corresponds for a particular amino acid. There are different codons coding for the same amino acid and hence they can be rare or abundant codons. In a way, codons coding for the same amino acid are selected differently during protein synthesis with respect to cell requirement [18]. This leads to codon bias which is known for its contribution in efficiency and accuracy of protein expression [19]. Owing to this, different codon positions are evolutionary selected to incorporate such codon bias in genome. We study how different codon positions are selected with identifying network motifs for 13 mtDNA genes as well as further extended our understanding by discerning network motifs between gene and other parts of the genome.

2 Evolution of variable sites

There are 42% variable sites observed which suggests not only almost half of the human mtDNA is mutated till date but also high mutation potential of mitochondrial genome. These variable sites are observed across all coding as well as non-coding genome regions. It is reported that there are various factors governing the mutations in the mitochondrial DNA, namely exceptionally high mutation potential produced by free oxygen radicals, less base-misincorporation rate that of nuclear DNA and its dependency on nuclear DNA repair machinery [20]. Different count of variable sites in different genome groups might indicate the evolutionary role of above mentioned factors in contributing mutation in mitochondrial genome. Also, 21% of variable sites are found to be commonly present in all genome groups, its suggests that there might be association of common factors in contributing mutations at common nucleotide positions. Analysis of variable sites in mtDNA facilitates the understanding of evolution and environmental adaptation which is directly affecting the diverse cellular processes including protein expression, regulation, and folding [21]. Cumulatively, variation in different nucleotide positions and their

different count in each genome groups indicates the diverse energy producing and spending mechanisms which might have evolved differently across human population.

3 Evolution of co-occurring variable sites

To explain the concept of nucleotide co-occurrence in the large genome, here we take an example of word collocation in literature. A word collocation is two or more words in the sentence or in a paragraph or elsewhere that often go together. Learning collocations is essential for making your English sound fluent and natural. These combinations just sound 'right' to native English speakers, who use them all the time. On the other hand, other combinations may be unnatural and just sound 'wrong'. For instance, (1) 'the fast train fast food' is natural to native speaker whereas 'the quick train quick food' is not natural. (2) 'a quick shower a quick meal' is natural to native speaker whereas 'a fast shower a fast meal' is not natural. Likewise, extending this fact words can co-occur together in the sentence and can be conserved together to form the proper meaning (Supplementary Fig. S5). There are several different types of collocation made from combinations of verb, noun, adjective etc. e.g., (adverb + adjective) or (adjective + noun). These collocations have helped in evolution of different languages.

Similarly, we define nucleotide co-occurrence where two nucleotides are co-occurring together. We find that the pairs of CO are observed to be members of single as well as two DNA segments, suggesting that co-occurrences might not only be the outcome of positive selection of individual variable site pair together but also have occurred as the result of mtDNA polygenic adaptation. Positive selection at divergent level implicates that the proportion of co-occurring nucleotide pairs are positively selected in one gene, but also experienced positive selection of nucleotide pairs between genes viz. one variable site is selected from one gene and second is picked up from other gene [22]. The comprehensive understanding of selection and adaptation in humans, specifically in mtDNA still remains an arduous. Many studies have reported that human evolutionary history believes to rely on selection of standing variation and polygenic adaptation [23]. When substitution of one nucleotide residue affects the evolution of other nucleotide residue, the nucleotide residues will, by definition, co-evolve and co-occur in genome sequences. Although co-evolutionary processes present at nucleotide residues in genomic sequences or at amino acids in protein sequences are clearly quite complicated. But, their potential reflection as molecular fossils to determine evolutionary history of these genomic sequences as well as interactions between genes makes understanding nucleotide co-occurrence in genomic sequences of extreme important. Ultimately, it is to be expected that analysis of the relationship between evolutionary events and gene function will lead to improved utility of such analyses in predictions of gene function and co-evolution of nucleotide residues in a single as well as multiple genes, *i.e.,* functional genomics. It is reported that there are low possibility of co-evolutation in mtDNA, the quasi simultaneous occurrence of deleterious amino acid changes and compensatory mutation or the occurrence of a deleterious mutation in a genetic environment characterized by a pre-existing compensatory background [24].

4 Selective role of Coding DNA

In order to find out, the role of coding DNA across mtDNA we calculate how many CO pairs are formed between different DNA segments and are atleast present in two genome groups. The data is represented in Table 2 in the manuscript. We find that most of these CO pairs are led by polypeptide genes. It is observed that there are a larger number of CO pairs formed between polypeptide genes. Also, these polypeptide genes tend to form more number of CO pairs with RNA region and it has very less affinity towards non-coding region. We also note with this is that non-coding region also have less propensity to form CO pairs between themselves.

5 Glossary

1. Genome: The genetic complement of an organism, including all of its GENES, as represented in its DNA, or in some cases, its RNA.
2. Mitochondria: Semiautonomous, self-reproducing organelles that occur in the cytoplasm of all cells of most, but not all, eukaryotes.
3. Sequence: The sequence of PURINES (A, G) and PYRIMIDINES (T, C) in nucleic acids and polynucleotides. It is also called nucleotide sequence.
4. Evolutionary Genomics: The genome of an organism is its complete set of DNA. The genomic sequences, and partical sequences, of organisms from several species can be used to study how genomes change during evolution. Evolutionary genomics is concerned with any question that can be asked about the evolution of genomes.
5. Codon: A set of three nucleotides in a protein coding sequence that specifies individual amino acids or a termination signal (CODON, TERMINATOR). Most codons are universal, but some organisms do not produce the transfer RNAs (RNA, TRANSFER) complementary to all codons. These codons are referred to as unassigned codons (CODONS, NON-SENSE).
6. Codon Bias: It refers to differences in the frequency of occurrence of synonymous codons in coding DNA.
7. Sequence mutation: In biology, a mutation is the permanent alteration of the nucleotide sequence of the genome of an organism, virus, or extrachromosomal DNA or other genetic elements.
8. Synonymous Mutation (silent): A nucleotide substitution resulting in a codon specifying the same amino acid as before (e.g., AAA to AAG, both code for lysine). The number of substitutions per synonymous site for any pair of sequences is K_s.
9. Non-synonymous Mutation: A substitution that alters a codon and causes a change in amino acid residue. The number of substitutions per nonsynonymous site for any pair of sequences is K_a. As nonsynonymous substitutions result in a biological change in the organism, they are subject to natural selection.
10. D-Loop: The mtDNA control region is an area of the mitochondrial genome which is non-coding DNA. It is the most polymorphic region of the human mtDNA genome [3].

11. Genetic drift: the random change in allele frequency that is caused by random variation in individual reproduction.
12. Transcription: the first step in the expression of a gene. It is the process via which DNA is enzymatically copied, by RNA polymerase, to produce a complementary RNA strand.
13. Positive selection: selection for beneficial mutations.

References

[1] Moritz, C. Applications of mitochondrial dna analysis in conservation: a critical review. *Mol Ecol.* **3**, 401-411 (1994).

[2] Taylor, R.W. & Turnbull, D. M. Mitochondrial dna mutations in human disease. *Nat Rev Genet.* **6**, 389-402 (2005).

[3] Schon, E.A., DiMauro, S., & Hirano, M. Human mitochondrial dna: roles of inherited and somatic mutations. *Nat Rev Genet.* **13**, 878-890 (2012).

[4] Koonin, E.V. Orthologs, paralogs, and evolutionary genomics 1. *Annu Rev Genet.* **39**, 309-338 (2005)

[5] i Cancho, R.F. & Solé, R. V. The small world of human language. *Proc R Soc Lond B* **268(2)**, 2261-2265 (2001).

[6] Liu, H. & Cong, J. Language clustering with word co-occurrence networks based on parallel texts. *Chin Sci Bull.* **58**, 1139-1144 (2013).

[7] Faust, K. et al. Microbial co-occurrence relationships in the human microbiome. *PLoS Comput Biol.* **8(7)**, e1002606 (2012).

[8] Wuchty, S. & Almaas, E. Evolutionary cores of domain co-occurrence networks. *BMC Evol Biol.* **5**, 1 (2005).

[9] Tatusov, R.L., Koonin, E.V. & Lipman, D.J. A genomic perspective on protein families. *Science* **278**, 631-637 (1997).

[10] Wang, Z. et al. A protein domain co-occurrence network approach for predicting protein function and inferring species phylogeny. *PloS One* **6(3)**, e17906 (2011).

[11] Du, X. et al. Networks of genomic co-occurrence capture characteristics of human influenza a (h3n2) evolution. *Genome Research* **18**, 178-187 (2008).

[12] Deng, L. et al. Network of co-mutations in ebola virus genome predicts the disease lethality. *Cell Research* **25**, 753 (2015).

[13] Milo, R. et al. Network motifs: simple building blocks of complex networks. *Science* **298**, 824-827 (2002).

[14] Mangan, S. & Alon, U. Structure and function of the feed-forward loop network motif. *Proc Natl Acad Sci.* **100**, 11980-11985 (2003).

[15] Alon, U. Network motifs: theory and experimental approaches. *Nat Rev Genet.* **8**, 450-461 (2007).

[16] Han, J.D. et al. Evidence for dynamically organized modularity in the yeast protein-protein interaction network. *Nature* **430**, 88-93 (2004).

[17] Jeong, H. et al., The large-scale organization of metabolic networks. *Nature* **407**, 651-654 (2000).

[18] Chaney, J.L. & Clark, P.L. Roles for synonymous codon usage in protein biogenesis. *Annu Rev Biophys.* **44**, 143-166 (2015).

[19] Hershberg, R. & Petrov, D.A. Selection on codon bias. *Annu Rev Genet.* **42**, 287-299 (2008).

[20] Balaban, R.S., Nemoto, S. & Finkel, T. Mitochondria, oxidants, and aging. *Cell* **120**, 483-495 (2005).

[21] Hockenberry, A.J., Sirer, M.I., Amaral, L.A. & Jewett, M.C. Quantifying position-dependent codon usage bias. *Mol Biol Evol.* **126**, (2014).

[22] Enard, D., Depaulis, F. & Crollius, H.R. Human and non-human primate genomes share hotspots of positive selection. *PLoS Genet.* **6**, e1000840 (2010).

[23] Fu, W. & Akey, J.M. Selection and adaptation in the human genome. *Annu Rev Genomics Hum Genet.* **14**, 467-489 (2013).

[24] Castellana, S., Vicario, S. & Saccone, C. Evolutionary patterns of the mitochondrial genome in metazoa: exploring the role of mutation and selection in mitochondrial protein-coding genes. *Genome Biol Evol.* **3**, 1067-1079 (2011).

Supplementary Figures

Codon based co-occurrence network motifs in human mitochondria

Pramod Shinde[1], Camellia Sarkar[1], Sarika Jalan[1,2*]

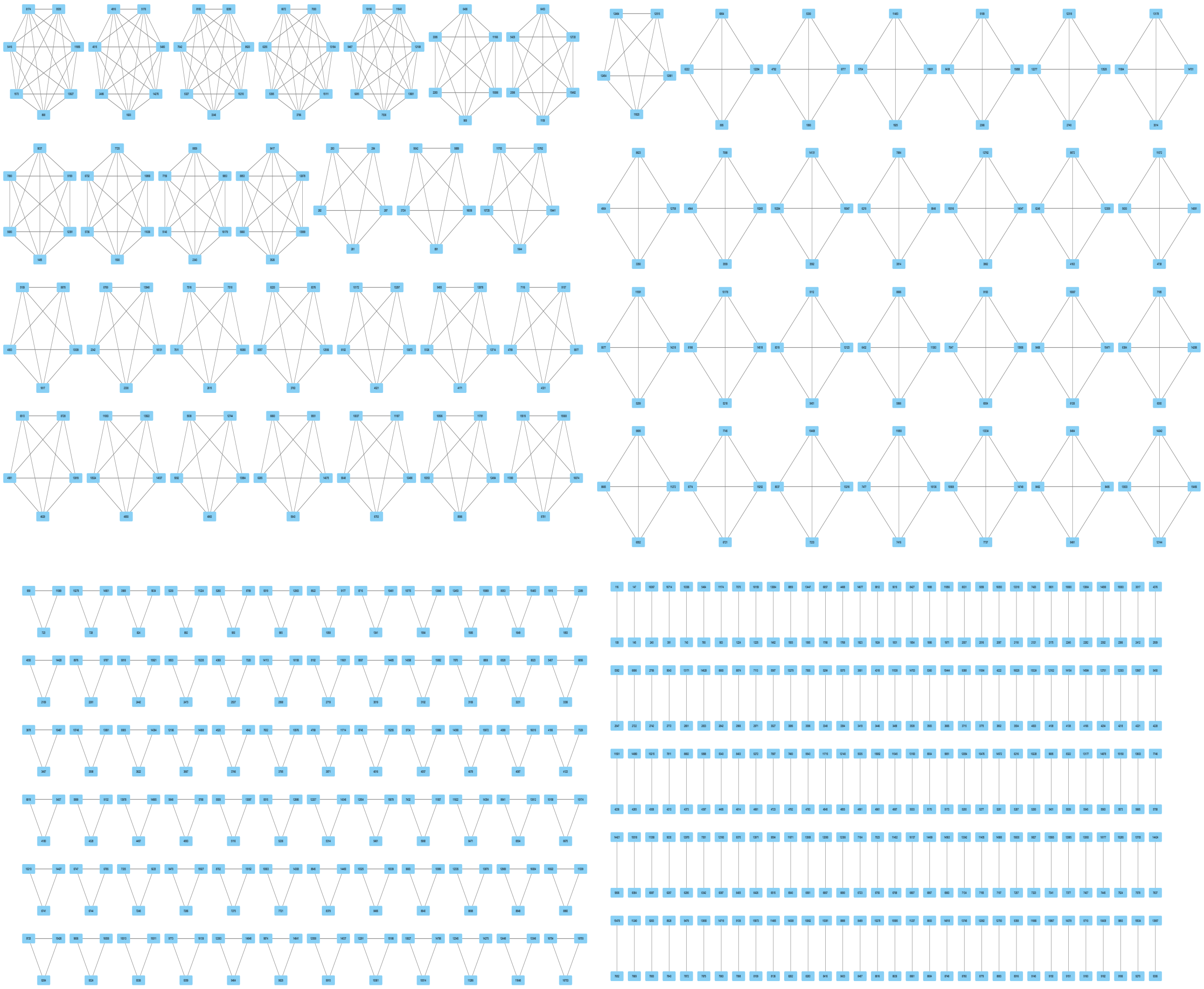

Supplementary Figure S1: **Schematic of nucleotide co-occurrence network.**

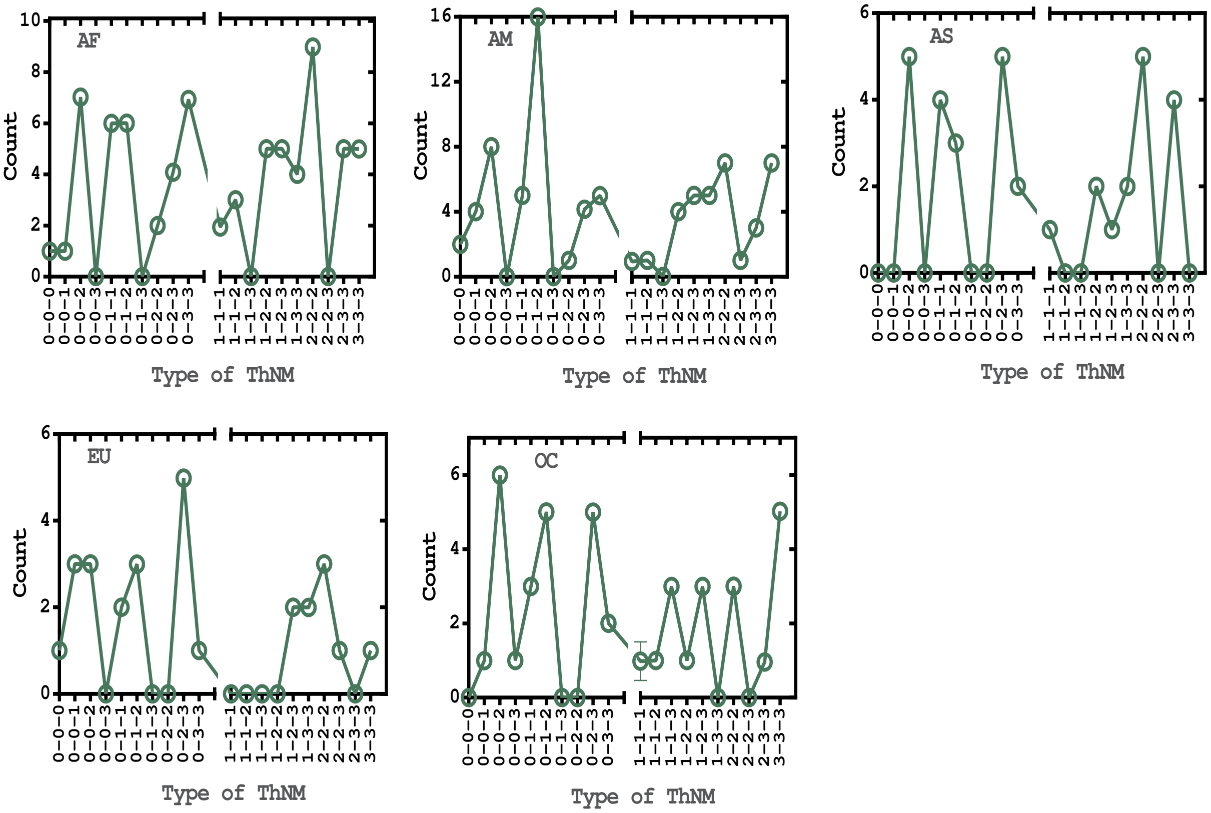

Supplementary Figure S2: **Distribution of ThNMs found in the randomized networks among different genome groups.** There are 20 possible ThNMs. These motifs are made up of different codon positions such as 1, 2 and 3 whereas 0 in motif represents the nucleotide position of non-codon region. Circles denote motif count in randomized networks.

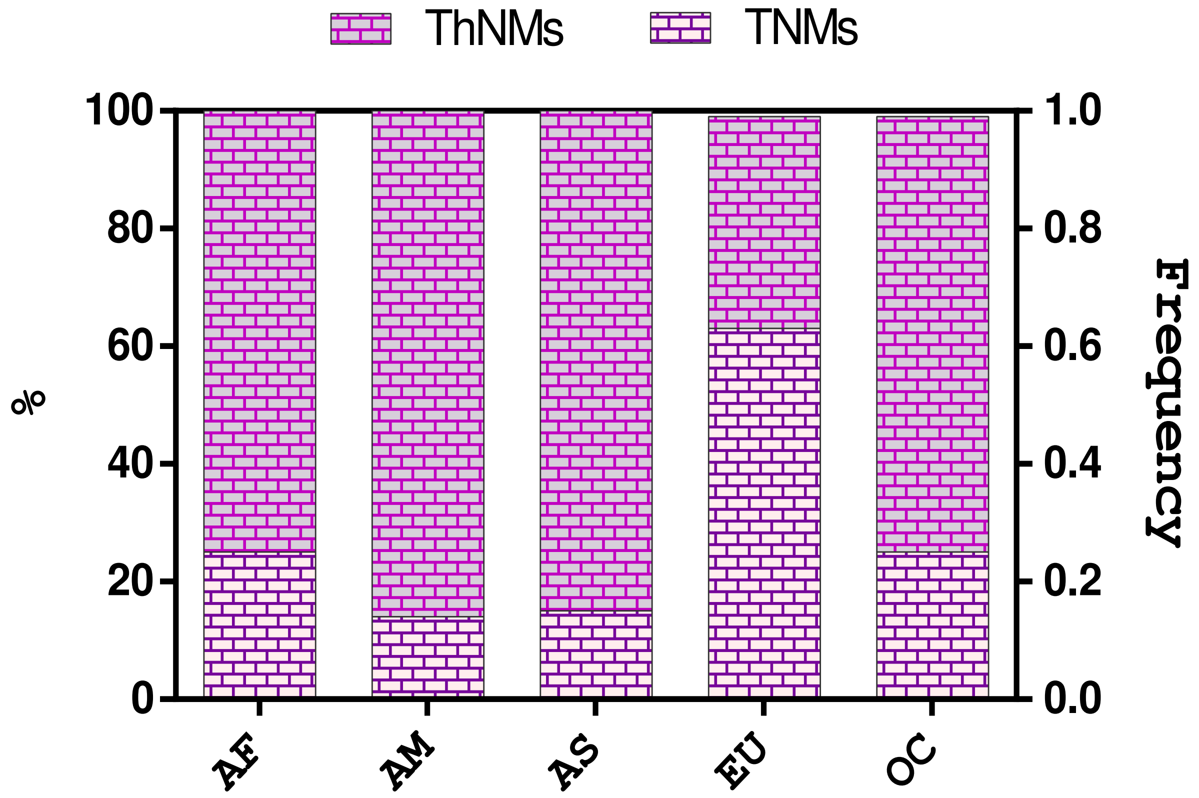

Supplementary Figure S3: **Frequency of TNMs and ThNMs among genome groups.**

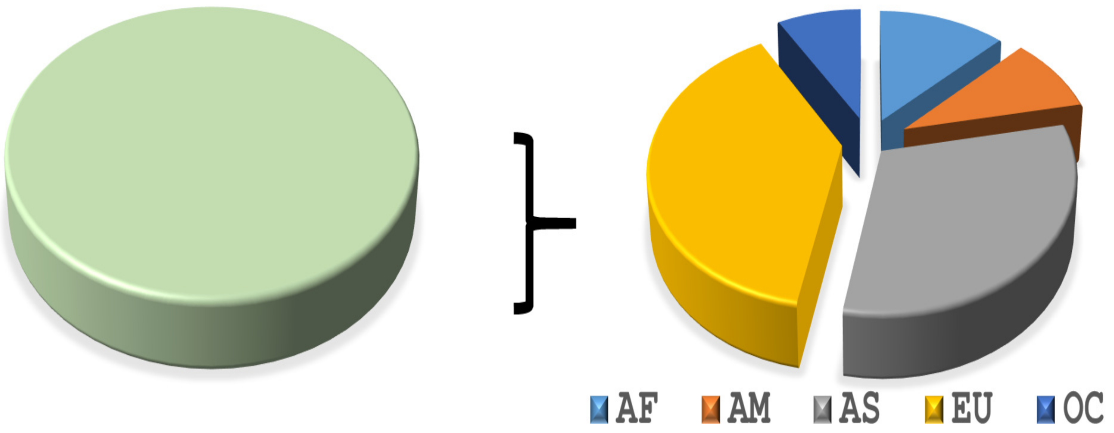

Supplementary Figure S4: **Mitochondrial genome data processing.** Total mtDNA samples are divided into five genome groups namely, Asia (AS), Africa (AF), America (AM), Europe (EU) and Oceania (OC).

"The quick hairy lion jumped over the lazy sloth."

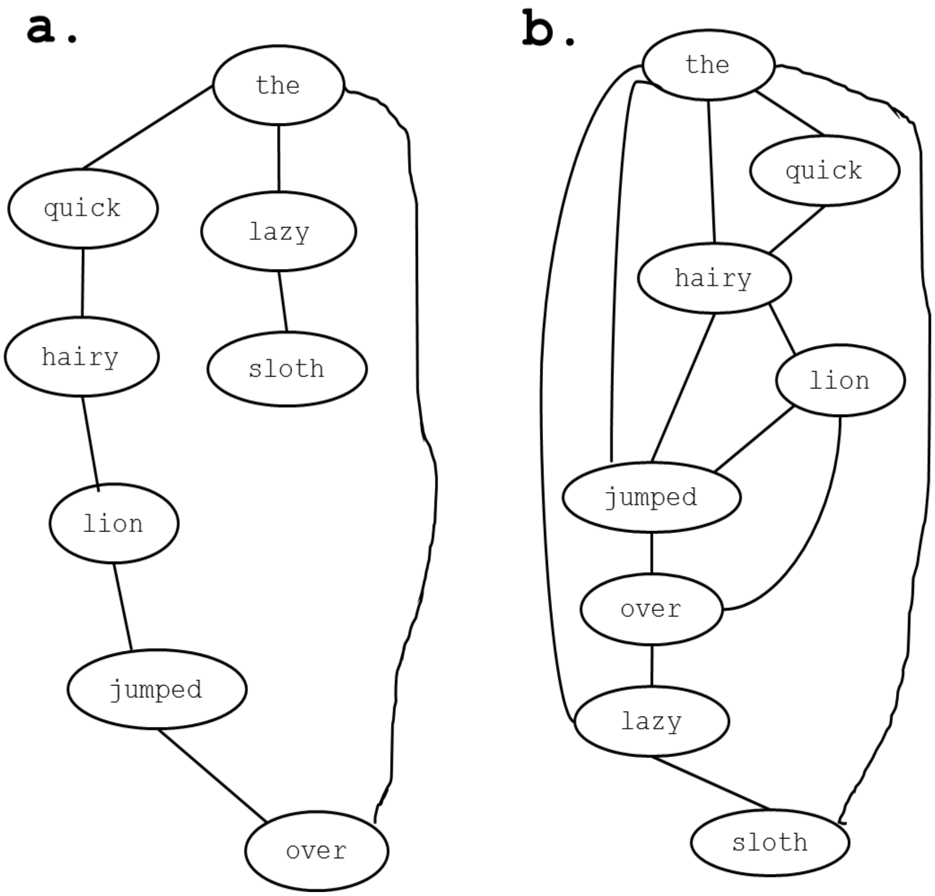

Supplementary Figure S5: **Schematic explaining the word collocations in the sentence.** Every word is associated with the word coming to its next (in a) or words can be associated with other words if they form some meaning or they just look natural (in b).

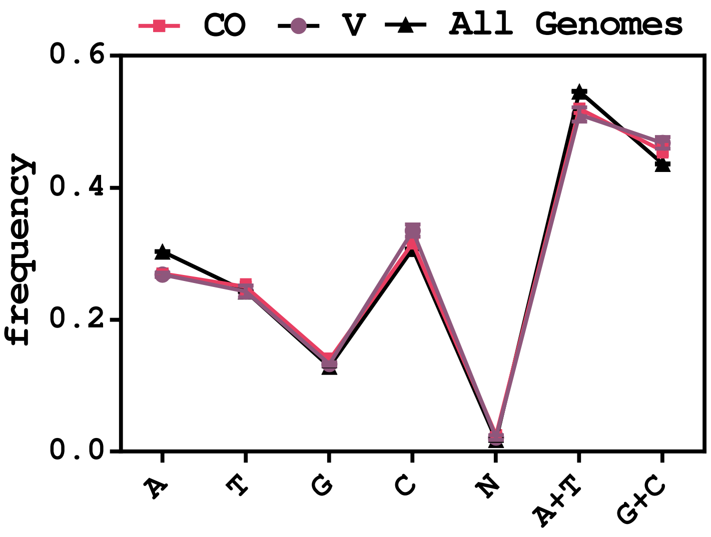

Supplementary Figure S6: **Frequency of each nucleotide among genome groups.**

Supplementary Tables

Codon based co-occurrence network motifs in human mitochondria

Pramod Shinde[1], Camellia Sarkar[1], Sarika Jalan[1,2*]

	Count	DNA segments
Polypeptide genes	13	*ATP6, ATP8, COX1, COX2, COX3, CYTB, ND1, ND2, ND3, ND4, ND4L, ND5, ND6*
tRNAs	22	ala, arg, asn, asp, cys, gln, glu, gly, his, Ile, leu (2 genes), lys, met, phe, pro, ser (2 genes), thr, trp, tyr, val
rRNAs	2	12s rRNA, 16s rRNA
Non-Coding	3	D-Loop, *oriL*, miscellaneous

Supplementary Table S1: **Genomic segments in human mtDNA.** There are total 40 DNA segments which make mtDNA viz. 13 protein coding or polypeptide genes, 22 tRNA genes, two rRNA genes and non-coding region comprises of D-Loop, oriL, miscellaneous.

Gene	Protein	UniProtKb ID
ATP6	ATP synthase subunit A	P00846
ATP8	ATP synthase protein 8	P03928
COX1	Cytochrome C oxidase subunit 1	P00395
COX2	Cytochrome C oxidase subunit 2	P00403
COX3	Cytochrome C oxidase subunit 3	P00414
CYTB	Cytochrome B	P00156
ND1	NADH-ubiquinone oxidoreductase chain 1	P03886
ND2	NADH-ubiquinone oxidoreductase chain 2	P03891
ND3	NADH-ubiquinone oxidoreductase chain 3	P03897
ND4	NADH-ubiquinone oxidoreductase chain 4	P03905
ND4L	NADH-ubiquinone oxidoreductase chain 4L	P03886
ND5	NADH-ubiquinone oxidoreductase chain 5	P03915
ND6	NADH-ubiquinone oxidoreductase chain 6	P03923

Supplementary Table S2: **List of genes with their associated protein names and UniProtKb Ids.** Protein sequences of mitochondrial genes are extracted from UniprotKb (http://www.uniprot.org/; Jan 08, 2016) and these sequences are used to map gene start and end sites of individual genes.
